# The Retinoid-Related Orphan Receptor RORα Promotes Keratinocyte Differentiation via FOXN1

**DOI:** 10.1371/journal.pone.0070392

**Published:** 2013-07-29

**Authors:** Jun Dai, Yang Brooks, Karine Lefort, Spiro Getsios, G. Paolo Dotto

**Affiliations:** 1 Cutaneous Biology Research Center, Massachusetts General Hospital, Charlestown, Massachusetts, United States of America; 2 Department of Dermatology, Lausanne University Hospital, Epalinges, Switzerland; 3 Department of Dermatology, Northwestern University Feinberg School of Medicine, Chicago, Illinois, United States of America; 4 Department of Biochemistry, University of Lausanne, Epalinges, Switzerland; University of Birmingham, United Kingdom

## Abstract

RORα is a retinoid-related orphan nuclear receptor that regulates inflammation, lipid metabolism, and cellular differentiation of several non-epithelial tissues. In spite of its high expression in skin epithelium, its functions in this tissue remain unclear. Using gain- and loss-of-function approaches to alter RORα gene expression in human keratinocytes (HKCs), we have found that this transcription factor functions as a regulator of epidermal differentiation. Among the 4 RORα isoforms, RORα4 is prominently expressed by keratinocytes in a manner that increases with differentiation. In contrast, RORα levels are significantly lower in skin squamous cell carcinoma tumors (SCCs) and cell lines. Increasing the levels of RORα4 in HKCs enhanced the expression of structural proteins associated with early and late differentiation, as well as genes involved in lipid barrier formation. Gene silencing of RORα impaired the ability of keratinocytes to differentiate in an in vivo epidermal cyst model. The pro-differentiation function of RORα is mediated at least in part by FOXN1, a well-known pro-differentiation transcription factor that we establish as a novel direct target of RORα in keratinocytes. Our results point to RORα as a novel node in the keratinocyte differentiation network and further suggest that the identification of RORα ligands may prove useful for treating skin disorders that are associated with abnormal keratinocyte differentiation, including cancer.

## Introduction

The retinoid related orphan receptor RORα belongs to the steroid nuclear hormone receptor superfamily and functions as a transcription factor by binding as a monomer to the RORα responsive elements (ROREs) in the regulatory region of target genes [1], [2], [3]. In humans, there are four RORα isoforms (RORα 1–4), which differ only in their amino-terminal A/B domain and are generated by alternative promoter usage and exon splicing [1], [4]. RORα is expressed in a temporal and spatial-dependent manner during embryonic development, and is critical for lipid metabolism [5], [6], [7], inflammation, and differentiation of Purkinje neuronal cells, adipocytes, lymphocytes, osteoblasts, as reviewed in [2], [3].

RORα is widely expressed in normal epithelial tissues [8], including the epidermis [9], [10], and is often down modulated in epithelium-derived tumors [8]. Yet, very little is known about the functions of RORα in epithelial cell biology. In mice, RORα transcripts are highly expressed in the skin, specifically in suprabasal epidermal cells, sebaceous glands, and hair follicles [9]. Mice with homozygous disruption of the RORα gene exhibit sparse pelage, with a slow rate of hair re-growth after shaving, pointing to a possible role of RORα in epidermal differentiation [9].

The epidermis is a stratified epithelial tissue structure composed of a single basal layer of proliferating keratinocytes that become progressively more differentiated as they migrate upwards and transit through the overlying spinous, granular, and cornified stratum layers [11]. The keratinocyte life cycle is associated with well-characterized molecular and biochemical changes. Keratinocytes at each stage express signature structural and signaling molecules [12]. For example, keratinocytes switch from expressing the keratin 5/14 pair in the basal layer to keratins 1/10, followed by involucrin, in the suprabasal layers. At later stages of differentiation in the granular layer, keratinocytes begin to express filaggrin and loricrin and produce lipid granules containing cholesterol, free fatty acids, and ceramides [13].

A well-controlled transcription network is critical for the balanced transition of keratinocytes from one stage to the next and the long-term maintenance of skin homeostasis [11], [14]. The initial switch from basal to spinous differentiation is controlled by Notch and p63 signaling pathways, which interplay with each other and with other transcription factors, such as interferon regulatory factor 6 (IRF6), AP2, and Kruppel-like factor 4 (KLF4) [15], [16], [17], [18], [19], [20] The early stage of differentiation is also regulated by FOXN1, a member of forkhead transcription factor family, via the interplay with the PKC and Akt signaling pathways [21], [22].

Epidermal keratinocytes provide an excellent experimental system to study intrinsic growth/differentiation controls of epithelial cells. Well defined culture conditions make it possible to study primary keratinocytes of human and mouse origin in vitro [23]. Experimental systems, such as organotypic culture and grafting of cultured keratinocytes, recapitulate the complex cell-cell interactions that occur in the epidermis *in vivo* [17], [24], [25]. Applying gain- and loss-of function approaches to the above experimental system, we show that RORα is a key integral element of the pro-differentiation network in epidermal keratinocytes, functioning as an upstream regulator of the FOXN1 gene and genes involved in epidermal lipid/barrier functions.

## Results

### 1) RORα4 Isoform Expression is up-regulated during Differentiation in Human Keratinocytes, and Down-regulated in Keratinocyte-derived Skin Cancer

**Figure 1:**
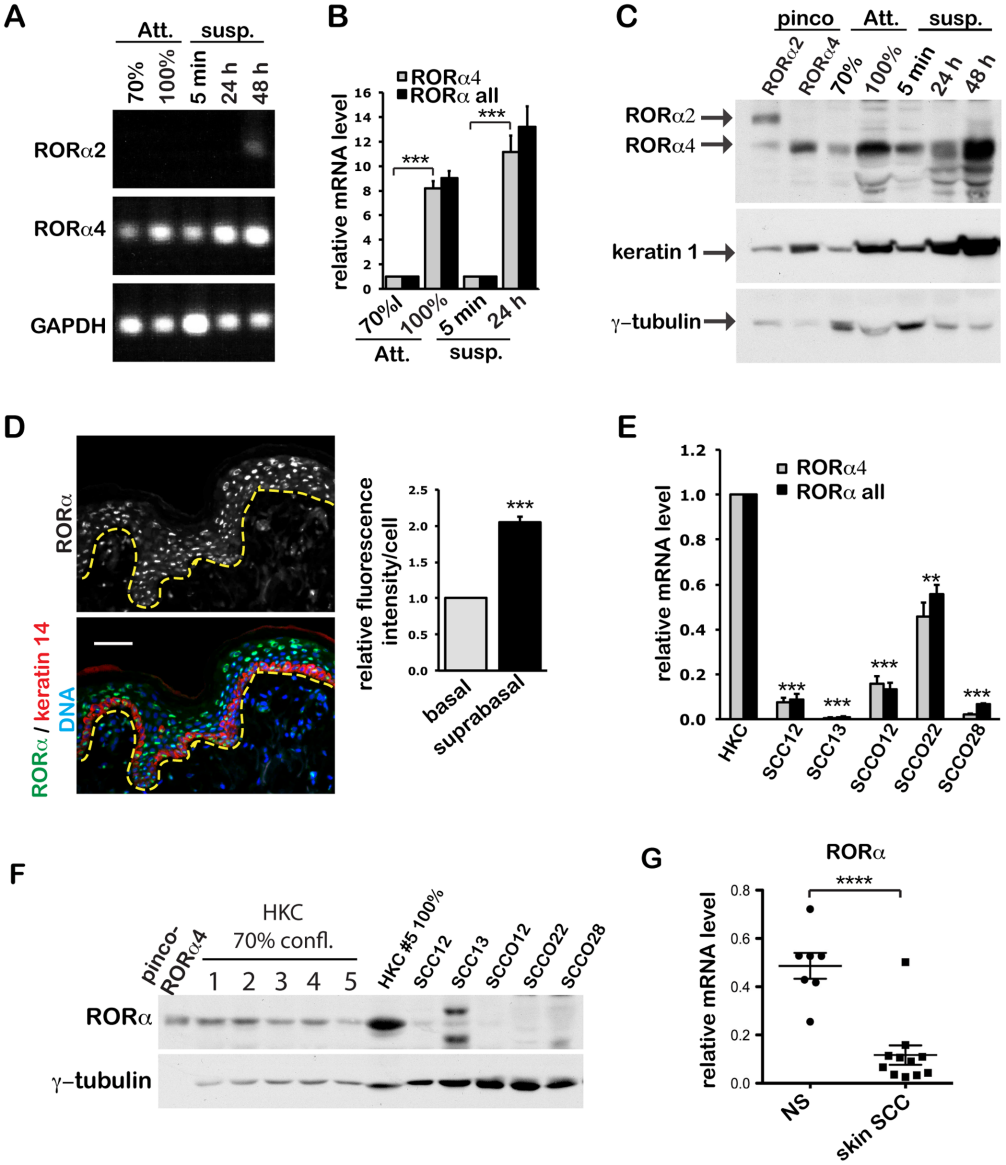
RORα4 expression is induced upon keratinocyte differentiation and down-regulated in keratinocyte-derived skin SCC. (**A**) RT-PCR analysis of RORα 2 and 4 expression in primary human keratinocytes (HKCs) cultured under growing (70% confluence), or differentiating conditions as induced by 100% confluence for 1 week or suspension culture on poly HEMA-coated dishes for 24 or 48 h. GAPDH was used as an internal control. (**B**) Real time qRT-PCR analysis of HKCs with primers specific for RORα4 or for all RORα isoforms. Values are normalized to 36B4, and presented as mean fold-change over control ± S.E.M., ***, p<0.001, N = 4 batches of HKCs. (**C**) Western blot analysis of RORα in HKCs under growing versus differentiating conditions. Lysates from sub-confluent HKCs infected with RORα2 or RORα4 expressing retroviruses were used as an indicator of the migration rate of the two isoforms in gels. Keratin 1 and γ-tubulin were used as indicators of differentiation and loading control, respectively. (**D**) Immunofluorescence analysis of RORα expression in human skin. Frozen sections (8 µm) of normal human skin were co-stained with antibodies against RORα (green) and keratin 14 (red). DNA was with counterstained with Hoechst (blue). Images are representatives of several independent fields (see more in Fig. S1), bar = 50 µm Fluorescence intensity of RORα/cell was quantified in 100 cells (from 3 human skin samples) in K14 positive basal versus negative (suprabasal) keratinocytes. Data are presented as mean fold-change of fluorescence signal/cell over signal/cell in basal layer ± S.E.M., ***, p<0.001, N = 3 human skin samples. (**E–F**) Real time qRT-PCR (E) and western blot analysis (F) of RORα in HKCs (N = 5 batches), in parallel with keratinocyte-derived skin SCC12 and SCC13, as well oral SCCO12, SCCO22, and SCCO28 cell lines. Primers specific for RORα4 or all RORα isoforms were used for the RT-PCR analysis. Values are normalized to 36B4, and presented as mean fold-change over HKCs ± S.E.M. **, p<0.05 ***, p<0.001, N = 3. (**G**) Real time qRT-PCR analysis of RORα expression in clinically occurring skin SCC tumors versus normal epidermis (NS), ****, p<0.0001.

To determine which RORα isoforms are expressed in keratinocytes, conventional RT-PCR analysis was performed using primers specific for the 4 individual isoforms. We found that RORα4 was the predominant isoform present in human primary keratinocytes (HKCs) cultured under growing conditions, and upon induction of differentiation by high density or suspension cultures [28] (Fig. 1A). While RORα1 and RORα3 were detectable in none of these conditions, low expression of RORα2 mRNA was detected only in cells induced to differentiate by 48-hour culture in suspension. Real time qRT-PCR analysis using a set of primers common for all RORα isoforms, as well as one specific for RORα4, showed a similar induction of RORα mRNA expression upon differentiation (Fig. 1B).

The RORα2 and RORα4 proteins have different size and electrophoretic mobility. The two proteins were clearly distinguished by immunoblot analysis of HKC extracts infected with retroviruses expressing RORα2 or RORα4 cDNAs. Consistent with the mRNA results, endogenous RORα2 protein was barely detectable, while RORα4 expression was most prominent and increased with the differentiation marker keratin 1 (Fig. 1C). Immunofluorescence staining of intact human skin showed that RORα was present in both basal and suprabasal layers (Fig. 1D, Fig. S1). Nevertheless, the fluorescence intensity of RORα signal in the suprabasal layers was significantly stronger than in the keratin 14-positive cells of the basal layer (Fig. 1D). In addition, RORα staining was mainly concentrated in the nucleus, consistent with its function as transcription factor.

SCC development is associated with altered keratinocyte differentiation [14], [29]. RORα4 mRNA and protein levels were reduced in a panel of skin squamous cell carcinoma (SCC) cell lines when compared with a panel of normal HKCs (Fig. 1E, F). Real time qRT-PCR analysis also showed a significant down-modulation of RORα expression in a set of clinically occurring cutaneous SCCs when compared with normal epidermis (Fig. 1G). Immunofluorescence staining of SCC specimens revealed a decrement of RORα expression, when compared with surrounding relatively normal epidermis (Fig. S2). These results further indicate that RORα may play an important role in maintaining skin homeostasis.

### 2) RORα Promotes Differentiation of Human Keratinocytes

**Figure 2:**
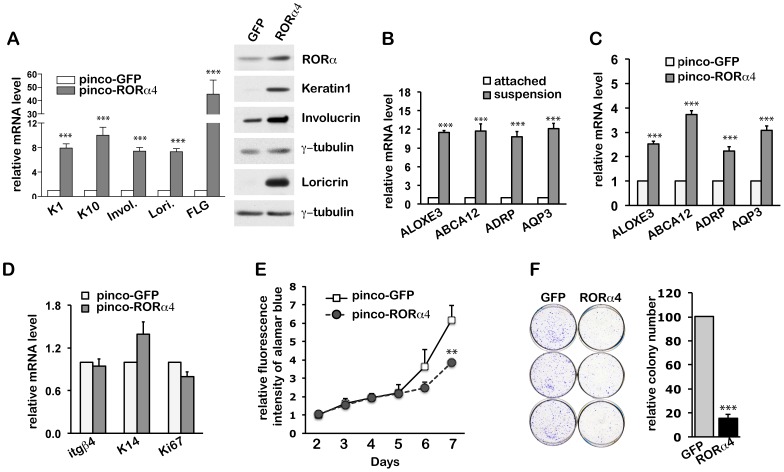
Increased expression of RORα4 triggers differentiation and inhibits proliferation of HKCs. (**A**) Real time qRT-PCR and western blot analysis of differentiation markers in HKCs at 72 h after infection with retroviruses expressing RORα4 or GFP alone. The mRNA level of each gene is normalized for 36B4 expression, and presented as mean fold-change over control ± S.E.M., ***, p<0.001, N = 3. (**B**) RT-PCR analysis of the indicated genes in HKCs under growing versus differentiating conditions as induced by suspension culture for 24 hours. Results are presented as mean fold-changes over control ± S.E.M., ***, p<0.001, N = 3. (**C–D**) Real time qRT-PCR analysis of the indicated genes in samples from (A). Data are presented as mean fold-change over controls ± S.E.M., ***, p<0.001, N = 3. (**E**) Alamar blue cell proliferation assay of HKCs in response to elevated RORα4 expression. Data are presented as mean fold-change of fluorescence intensity ± S.E.M. over control cells at day 2 after infection. **, p<0.01, N = 3. (**F**) Clonogenicity assay of HKCs in response to increased RORα4 expression. HKCs expressing RORα4 or GFP alone were analyzed for colony formation after 9 days of culture. Data are presented as mean percentage-change ± S.E.M. over control cells. ***, p<0.001, N = 3.

To determine whether RORα4 plays a role in keratinocyte differentiation, we overexpressed RORα4 in HKC at a level similar to that of the endogenous protein induced upon differentiation (Fig. 1C). At 72 h after infection, increased RORα4 expression significantly induced the expression of early and intermediate differentiation markers (keratin 1/10 and involucrin), as well as granular layer markers (loricrin and filaggrin) at both mRNA and protein levels (Fig. 2A).

The late stage of keratinocyte differentiation is associated with changes in cellular metabolism, in particular lipid-related, that are essential for epidermal outer layer formation [13]. A number of epidermal barrier-related genes, including ALOXE3, ABCA12, ADRP (adipose-differentiation related protein), and AQP3 (aquaporin 3) [30], [31], [32], [33], were up regulated in HKCs induced to differentiate by suspension culture (Fig. 2B), as well as by increased RORα4 expression (Fig. 2C), implicating RORα in this aspect of the keratinocyte differentiation program.

Increased RORα4 expression exerted no significant effect on markers of the basal proliferative compartment (Fig. 2D). However, Alamar blue density assay showed that increased RORα4 expression caused growth inhibition in HKCs from day 6 after transduction (Fig. 2E). The fraction of keratinocytes with clonal growth capability was also significantly reduced after prolonged culture (Fig. 2F). These results suggest that increased expression of RORα4 is sufficient to promote the differentiation program while reducing the long term growth potential of HKCs.

### 3) RORα is Essential for Keratinocyte Differentiation

**Figure 3:**
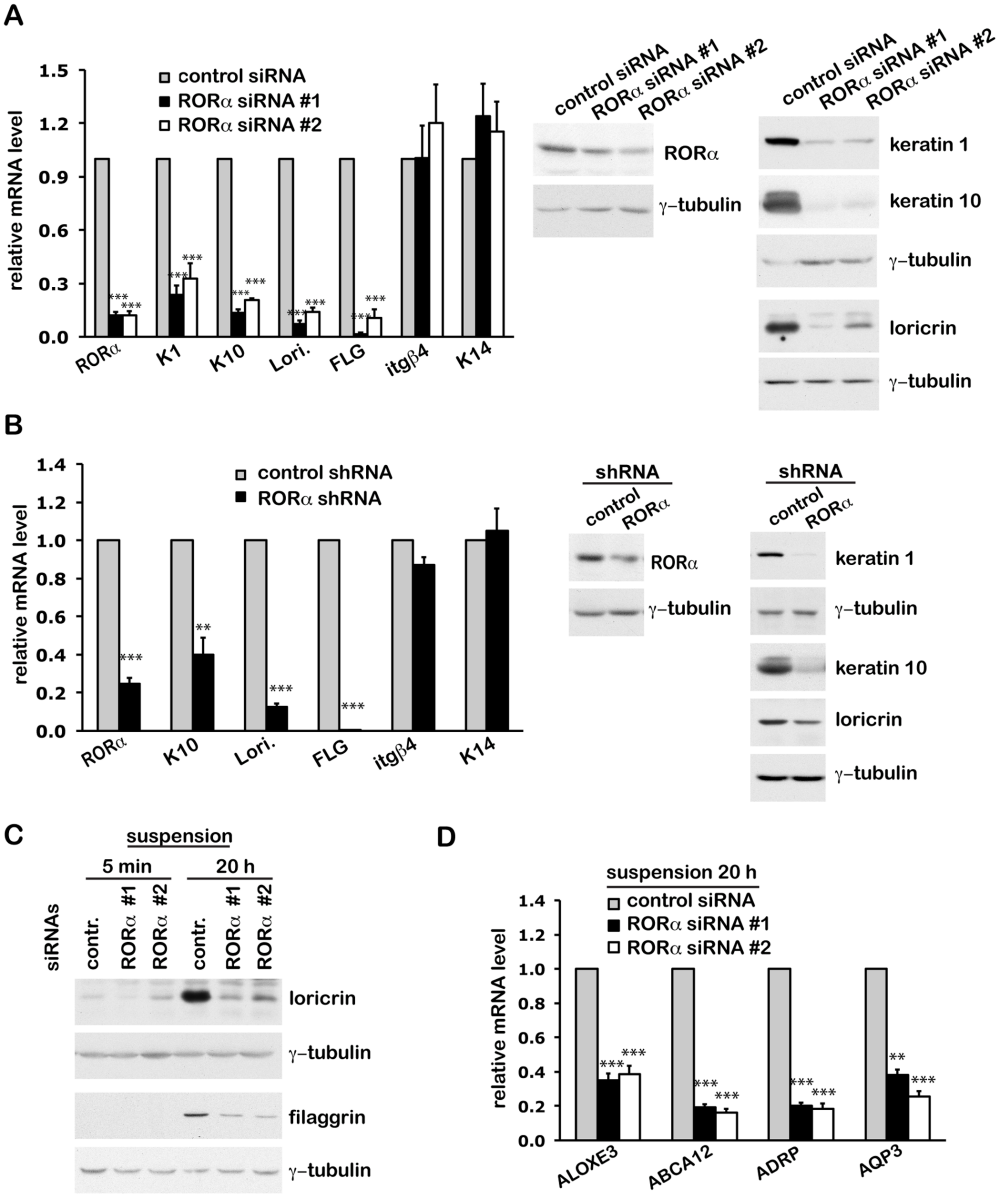
Silencing of RORα expression inhibits keratinocyte differentiation. (**A**) Real time RT-PCR and western blot expression analysis of the expression of indicated genes in HKCs transfected with two separate siRNAs against all RORα isoforms or control siRNA. Cells were harvested at day 4 after transfection when they reached 100% confluence. mRNA levels were normalized for 36B4, and presented as mean fold-change over control ± S.E.M. ***, p<0.001, N = 3. (**B**) Real time qRT-PCR and western blot analysis of indicated gene products in HKCs infected with lentiviruses expressing control shRNA or an shRNA against RORα. Cells were harvested 4 days after infection. Values are presented as mean fold-change over control ± S.E.M. **, p<0.01, ***, p<0.001, N = 3. (**C**) Western blot analysis of loricrin and filaggrin expression in HKCs transfected with control or RORα siRNAs. Seventy-two hours after transfection, sub-confluent cells were re-plated onto poly-HEMA coated plates, and harvested after 20 h culture in suspension. (**D**) Real time qRT-PCR analysis of the indicated lipid/epidermal barrier related genes in HKCs plus/minus RORα knockdown as in (C). Values are presented as mean fold-change over control ± S.E.M. **, p<0.01, ***, p<0.001, N = 3.

We next investigated whether RORα is required for normal differentiation, using a loss of function gene silencing approach. Two separate siRNAs and a lenti-viral shRNA that target all RORα isoforms were able to efficiently knock down RORα expression at both mRNA and protein levels (Fig. 3A, B). Although RORα silencing had no effect on the expression of basal layer markers like integrin β4 and keratin 14, it significantly reduced the expression of outer differentiation markers at mRNA and protein levels (Fig. 3A, B). For better evaluation of late differentiation, HKCs at growing density (72 h after siRNA transfection) were forced to differentiate by culture in suspension (Fig. 3C, D). RORα silencing decreased significantly the expression of loricrin, filaggrin (Fig. 3C) and epidermal barrier-related genes (Fig. 3D), indicating that RORα is broadly required for keratinocyte differentiation.

**Figure 4:**
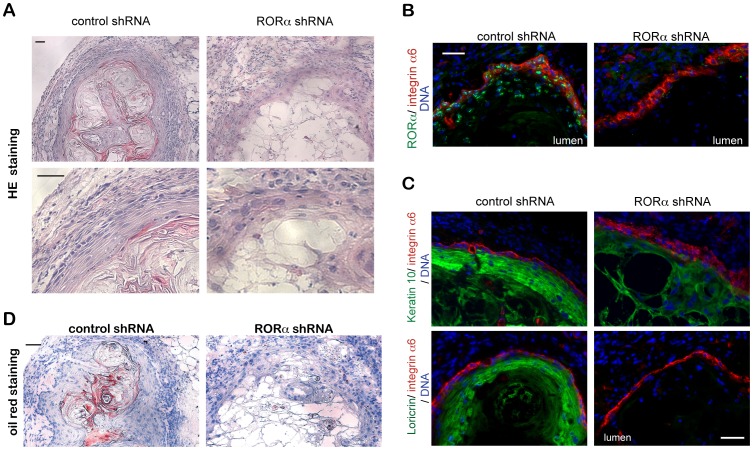
Silencing of RORα disrupts keratinocyte differentiation *in vivo*. HKCs stably infected with lentiviruses expressing shRNAs against RORα or control were injected intradermally into the back skin of NOD/SCID mice. To minimize the individual variation, control and RORα knockdown cells were injected in parallel in the right and left flank of mice. Resting nodules/cysts formed from HKCs were collected on day 8 after injection, and frozen sections were analyzed by (**A**) Hematoxylin & Eosin staining; (**B–C**) Immunofluorescence for the indicated proteins; or (**D**) oil red and hematoxylin staining to visualize lipids (red) and for nuclei (blue). Bar = 50 µm.

To determine whether RORα plays a similar regulatory function in vivo, HKCs infected with lenti-viruses expressing control or RORα shRNA were injected at the dermal-epidermal junction of immune-deficient NOD/SCID mice. As previously reported [17], [34], 8 days after injection, control cells formed well differentiated epidermal cysts, with pronounced granular and squamous layer formation. In contrast, RORα silencing led to structures without ordered layer formation and cornification (Fig. 4A). RORα expression, as assessed by immunofluorescence analysis, was much stronger in control cysts than in those formed by shRNA expressing keratinocytes, confirming the knockdown efficiency in vivo (Fig. 4B). Keratin 10 was strongly expressed in the well stratified and differentiated layers of control cysts, while weaker expression and association with loosely connected cells were found after RORα depletion (Fig. 4C, Fig. S3A). Loricrin was also strongly expressed in control cysts, while it was undetectable in the cysts formed by RORα knockdown keratinocytes (Fig. 4C, Fig. S3B). Oil red staining revealed that neutral lipids were accumulated in the stratum corneum of control cysts, but not of RORα knockdown cysts (Fig. 4D). Therefore, the in vivo cyst assays support the in vitro findings that RORα is required for normal keratinocyte differentiation, including the lipid layer formation program.

### 4) The Pro-differentiation Effects of RORα are Partially Mediated by FOXN1

**Figure 5:**
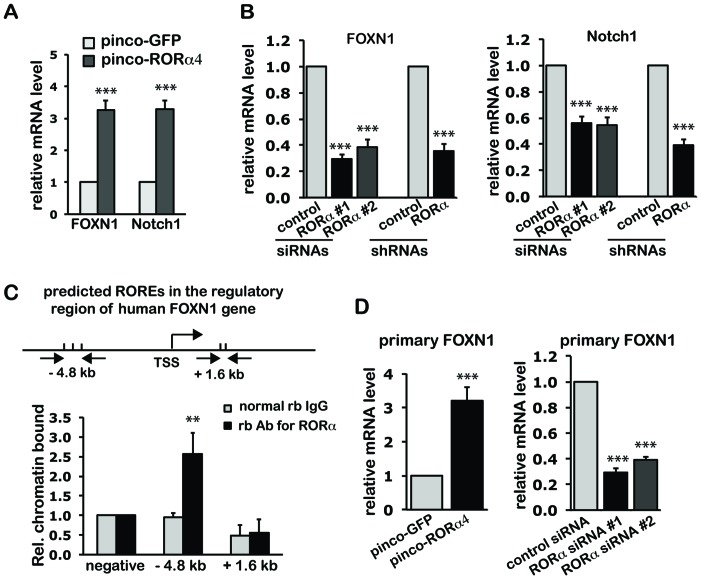
RORα positively regulates the expression of FOXN1. (**A–B**) HKCs with increased (A) or knocked-down (B) RORα expression were analyzed for expression of mature FOXN1 and Notch1 mRNA by real time qRT-PCR. Values are presented as mean fold-change over control ± S.E.M., ***, p<0.001, N = 3. (**C**) Chromatin immuno-precipitation (ChIP) analysis of RORα binding to the regulatory region of the FOXN1 gene. Mat-inspector software was used to search for RORα response elements (ROREs) within –6 kb and +2 kb from the transcription starting site (TSS) [Top]. Human epidermis was processed for ChIP assays utilizing rabbit antibodies specific for RORα or non-immune IgG control followed by PCR amplification of the indicated regulatory regions of the FOXN1 gene. The relative amount of precipitated DNA was calculated after normalization for total input chromatin, according to the following formula [59]: % total = 2^ΔCt^×5, where ΔCt = Ct (input) – Ct (immunoprecipitation), Ct, cycle threshold. Statistical significance of the results was determined by unpaired Student’s *t*-test, comparing the ratio RORα/IgG signal for each binding site relative to the one for the binding site at the RORE negative region. **, p<0.01, N = 3. (**D**) HKCs with increased or knocked down RORα expression were analyzed by qRT-PCR for primary FOXN1 transcript levels, using a primer corresponding to the first intron/exon junction. Samples were the same as described in (A–B). Values are presented as mean fold-change over control ± S.E.M., ***, p<0.001, N = 3.

Keratinocyte differentiation relies on an integrated transcriptional network, involving increased expression and activity of the FOXN1 and Notch1 genes. Real time qRT-PCR analysis showed that mRNA expression of FOXN1 and Notch1 was significantly induced by increased expression of RORα4 (Fig. 5A), and decreased by RORα silencing (Fig. 5B). In contrast, RORα level did not affect the expression of other transcription factors (p53, c-myc, and NF-κB) (Fig. S4). Mat-Inspector software (Genomatrix) was used to analyze the transcription regulatory region of these genes, spanning 6 kb of upstream and 2 kb of downstream sequence from the transcription start sites (TSS). Multiple consensus RORα response elements (ROREs) were found in both FOXN1 and Notch1 regulatory regions. Chromatin Immunoprecipitation (ChIP) analysis of human epidermis by real time RT-PCR showed binding of endogenous RORα to an upstream region of the FOXN1 gene containing a predicted RORα binding site (– 4.8 kb), but not to a downstream region containing another such site (+1.6 kb) (Fig. 5C). Despite the presence of predicted ROREs, ChIP assays failed to detect any significant binding of RORα to the Notch1 promoter region. Consistent with FOXN1 functioning as a direct RORα target, expression of the primary FOXN1 transcript, as detected by the primers corresponding to the first intron/exon junction, was similarly induced or blocked by modulation of RORα as the mature transcript (Fig. 5D).

**Figure 6:**
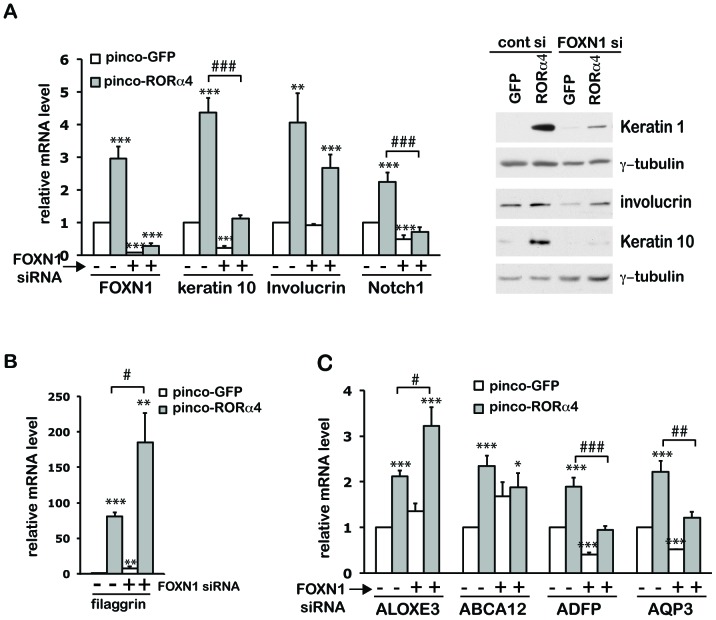
RORα-induced keratinocyte differentiation is partially mediated by FOXN1. (**A–C**) HKCs transfected with control siRNA or siRNA against FOXN1 were infected with retroviruses expressing GFP or RORα4 24 hours later. Samples were collected after additional 72 hours for qRT-PCR or western blot analysis of the indicated genes. qRT-PCR data are presented as mean fold-change over controls ± S.E.M. ***p<0.001, **p<0.01, *p<0.05, N = 3.

To assess whether FOXN1 functions as a mediator RORα in differentiation, we tested the impact of increased RORα4 expression in HKCs plus/minus FOXN1 silencing (Fig. 6). Induction of early differentiation markers keratin 1/10 and Notch1 by increased RORα expression was counteracted to a large extent by siRNA-mediated FOXN1 knockdown (Fig. 6A). FOXN1 silencing showed opposite enhancing effects on expression of filaggrin (Fig. 6B), consistent with previous findings that FOXN1 functions as repressor rather inducer of late keratinocyte differentiation markers [22]. Selective effects of FOXN1 knockdown were also observed with the epidermal barrier-related genes. Silencing of FOXN1 blocked the ability of RORα to induce ADFP and AQP3 expression, while causing no repression or even slight up-regulation of ALOXE3 and ABCA12 (Fig. 6C). These results establish FOXN1 as a direct RORα target and selective mediator of its function in differentiation.

## Discussion

The nuclear orphan receptor RORα plays a key role in embryonic development and a variety of physiological processes, such as lipid metabolism, bone formation, inflammation and T_H_17 cell differentiation [6], [35] [3]. In parallel with its inverse expression in normal keratinocytes and epidermis versus SCC cells and tumors, we have shown here that RORα plays an essential positive role in keratinocyte differentiation, with FOXN1 as a direct target and mediator. RORα4 is the major RORα isoform expressed in keratinocytes, and its expression is further induced upon differentiation at both mRNA and protein levels. In contrast to Notch1 and FOXN1, which promote early stages of keratinocyte differentiation and suppress the later ones [21], [36], [37], RORα4 is a positive determinant of both, with a role that extends to control of a group of genes that are involved in lipid synthesis, lipid delivery to lamellar bodies, lipid deposition/release from droplets, and water/glycerol transport [33], [38], [39], [40], [41], [42].

FOXN1 is a key player in the epidermal differentiation program [21], [22], hair follicle development [43], and skin pigmentation [44]. Moreover, we have previously reported that it functions as a determinant of benign versus malignant keratinocyte tumor development [27]. Little is known of the upstream factors that regulate FOXN1 expression in keratinocyte differentiation, except that it appears to be a direct negative target of the c-Jun transcription factor, and under opposite control of EGFR/ERK versus FGFR3 signaling [27], [37]. We have shown here that RORα can also directly bind to the regulatory region of the FOXN1 gene and induce its transcription. The findings are of functional importance, as silencing of FOXN1 prevented the ability of RORα4 to induce expression of a number of differentiation marker genes, as well as a few of the genes involved in epidermal/lipid barrier formation. Importantly, the ability of RORα to induce expression of Notch1, another key regulator of keratinocyte differentiation [29], was also prevented by the FOXN1 knock-down, consistent with a previous finding that FOXN1 is required to maintain Notch1 expression in the hair follicle matrix of mice [45]. In contrast to these genes, induction of filaggrin by RORα4 was enhanced rather than prevented by knocking down FOXN1, consistent with the already mentioned negative impact of this gene on late stages of differentiation [21], [37]. FOXN1 knockdown did not affect RORα4-induced expression of specific genes connected with the lipid barrier function like ALOXE3 and ABCA12. This indicates that RORα functions as inducer of keratinocyte differentiation through both FOXN1-dependent and -independent mechanisms. Such a conclusion is consistent with a recent report that filaggrin gene expression is positively controlled by RORα through an as yet undefined AP1-dependent mechanism [46].

A number of lipid products have been identified as natural or synthetic ligands or agonists/antagonists of RORα [47], including cholesterol sulfate [48], [49], [50], which is formed during squamous differentiation and may be involved in induction of RORα activity in the skin [46], [50]. In general, nuclear receptors function as intracellular sensors of various lipids (Evans 2004) and, in addition to being part of the structural component, lipids can serve as signaling molecules for nuclear receptors involved in establishment of the epidermal barrier [51]. Thus, by inducing lipid metabolizing genes, enhanced RORα expression has the potential of modulating lipid molecules involved in its own regulation and/or regulation of other nuclear hormone receptors with a role in the skin. In particular, ALOXE3, a target gene of RORα, encodes an epidermal specific lipoxygenase eLOX3, which acts as a hydroperoxide isomerase and generates specific types of epoxyalchols (hepoxilins) [52], [53]. Hypoxylins have been shown to bind and activate PPARα [38], and thus may mediate a cross talk with RORα function.

As reported in other epithelial tumors [8], we have found that RORα expression is down-regulated in keratinocyte-derived SCC cell lines and tumors. However, the causes of RORα low expression in SCCs are still unknown. One attractive possibility is that p53, often lost or mutated in skin SCCs, is positively controlling RORα, as already reported in colon cancer cell lines [54]. In this context, RORα acts as a positive feedback loop on p53 stability and pro-apoptotic functions [54], [55]. In our own work, we have also obtained evidence that RORα expression can be positively controlled by p53, even though this was not observed in all tested conditions and with different human keratinocyte strains (unpublished results).

Besides genetic heterogeneity of response, another level of complexity that still has to be deciphered in further studies is the connection between RORα and control of the circadian cycle. In fact, RORα is also known to play a role in the circadian cycle [56], and a cross-talk between this and cell cycle control is currently emerging as an important element of cancer susceptibility [57]. It has also been reported that the circadian clock temporally fine-tunes the self renewal potential of epidermal stem cells [58]. Thus, besides its clear role in keratinocyte differentiation, RORα may play additional functions in the skin, depending on growth/differentiation stages of keratinocytes and/or in response to multiple exogenous signals.

## Materials and Methods

### Ethics Statement

The animal study (protocol #: 2004N000170) was specifically approved by the Subcommittee on Research Animal Care (SRAC), which serves as the Institutional Animal Care and Use Committee (IACUC) in Massachusetts General Hospital. The animal study was carried out in strict accordance with the recommendations in the Guide for the Care and Use of Laboratory Animals of the National Institutes of Health. All surgery was performed under sodium pentobarbital anesthesia, and all efforts were made to minimize suffering.

### Cell Culture and Human Specimens

Primary human keratinocytes were isolated and cultured in serum-free keratinocyte-SFM medium (Gibco) supplemented with 30 µg/ml bovine pituitary extract (BPE) and 0.2 ng/ml rEGF on the collagen coated plates. Suspension induced differentiation was achieved by culturing HKCs on the poly (2-hydroxyethylmethacrylate) [poly-HEMA] (Sigma) coated Petri dishes for 24 or 48 hours. SCC13 and SCC12 cell lines were provided by Dr. J. Rheinwald (Brigham and Women’s Hospital), and the SCC O12, O22 and O28 cells were provided by Dr. J. Rocco (Massachusetts General Hospital). The information of skin SCC samples is described previously [17].

### Plasmids and Viruses

The retroviral pinco-Flag-RORα2 and pinco-Flag-RORα4 plasmids were generated by cloning the Flag-tagged full-length cDNAs of the two human RORα isoforms into the BamHI/EcoRI sites of the pinco-GFP vector. The cDNAs with restriction enzyme sites were generated from PCR using pcDNA-RORα2 and pCR-BluntII-TOPO-RORα4 plasmids (Open Biosystems) as templates, as well as the following primers 1) Forward for RORα4∶5′-GATTCCGGATCCGCCACCATGGACTACAAGGACGACGATGACAAGATGATGTATTTTGTGATCGCAGCG; 2) Forward for RORα2∶5′- GATTCCGGATCCGCCAC-CATGGACTACAAGGACGACGATGACAAGATGAATGAGGGGGCCCCAGGAGAC; 3) Reverse for both RORα2 and RORα4∶5′- GCTGCTGAATTCCTATTACCCATCAA-TTTGCATTGCTGG. The lentiviral MISSION shRNA against all RORα isoforms is obtained from Sigma (TRCN0000022154). Conditions for retro- and lenti-virus production and infection were as previously reported [26].

### Quantitative Real Time RT PCR and Microarray Analysis

For mRNA analysis, 500 µg of total RNA, isolated with RNeasy Mini QIAcube kit (Qiagen), was reversely transcribed into cDNA using the iScript cDNA synthesis kit (Bio-Rad, Hercules, CA, USA). The PCR procedure and primers for RORα 1–4 isoforms were as described previously (Pozo et al. 2004). qRT-PCR with SYBR Green detection (Roche Applied Science, Indianapolis, IN, USA) was performed on the Light Cycler 480 Real Time PCR instrument (Roche Applied Science), according to manufacturer’s instructions. Each sample was tested in triplicate, and results were normalized with the expression of the housekeeping 36β4 gene. The list of gene-specific primers for qRT-PCR is provided in Table S1.

### Fluorescence Microscopy and Histological Analysis

Frozen blocks of specimens were sectioned and fixed with 4% paraformaldehyde in PBS at room temperature for 30 min. After permeablization with 0.1% NP-40/PBS, slides were blocked with 5% normal donkey serum/PBS and incubated with the primary antibodies in the blocking buffer at 4°C overnight. After washes with 0.1% NP-40/PBS, slides were incubated with Alexa 488 (green)- or Alexa 594 (red)-conjugated secondary antibodies (Invitrogen, Grand Island, NY, USA), plus Hoechst (Invitrogen) for DNA detection. Fluorescence microscopy was carried with Nikon TE300 inverted fluorescence microscope. The primary antibodies include: rabbit anti-RORα (ab60134, Abcam, Cambridge, MA, USA), rat anti-integrin α6 (CD49f, Chemicon International Inc., Temecula, CA, USA), mouse anti-keratin 14 (ab9220, Abcam), rabbit anti-keratin 1 (PRB-149P, Covance, Dedham, MA, USA), rabbit anti-keratin 10 (PRB-159P, Covance), mouse anti-involucrin (I9018, Sigma, St. Louis, MO, USA), rabbit anti-loricrin (PRB-145P, Covance), and rabbit anti-Ki67 (ab16667, Abcam). For histological analysis, frozen sections were fixed with 10% formalin, and stained with Haematoxylin & Eosin (Protocols, kalamazoo, MI, USA). Bright field images were collected with Zeiss Axio Observer Z1 inverted microscope. The average fluorescence intensity of RORα signal in each cell was quantified by the Image J software.

### Western Blot Analysis

Conditions for immuno-blotting were as previously described, with the same antibodies listed for fluorescence microscopy, except rabbit anti-RORα (sc-28612, Santa Cruz Biotechnology Inc., Santa Cruz, CA, USA). As loading controls, membranes blotted with rabbit antibodies were incubated with blocking buffer containing 0.2% sodium azide and were re-probed with mouse anti-γ-tubulin (GTU-88, Sigma) antibody.

### Alamar Blue Assay

Cell proliferation was measured by the Alamar Blue assay (Invitrogen). HKCs were plated in triplicate on the 96-well collagen coated plates at a density of 1000 cells/well in 100 µl medium. At the specific time points, 5 µl of Alarma Blue reagent was added to the medium for 1 hour at 37°C. Fluorescence was monitored at 530–560 nm excitation wavelength and 590 nm emission wavelength on the Victor™ X3 Multilabel Plate Reader (Perkin Elmer, Salem, MA, USA).

### Clonogenicity Assay

HKCs were plated at a density of 1000 cells/well in the 6-well plates without collagen coating. Nine days later, cells were fixed with 100% ethanol for 10 min at RT, followed by staining with 0.1% crystal violet in 10% ethanol at RT for 1 hour. After washes in water, the plates were dried at RT overnight. Colonies were counted with the Image J software.

### SiRNA Transfection

Primary human keratinocytes were reversely transfected with 40 nM of predesigned siRNAs (Ambion-Invitrogen) for human RORα (s12103, s12105), FOXN1 (s16062, s16060), and control (4390844) using Hiperfect reagent (Qiagen), according to manufacture’s instructions.

### Cyst Assay

For *in vivo* cyst assays, HKCs infected with lenti-virus expressing control or RORα shRNAs were selected by puromycin 2 days after infection. After selection, cells were collected and admixed with matrigel (4∶1), followed by intra-dermal injection (2×10^6^ cells per spot) into the back skin of NOD/SCID mice (Taconic Farms Inc. Germantown, NY, USA), as previously described [17]. To minimize the individual animal variations, HKCs plus/minus RORα knockdown were injected in parallel in the right and left flank of the same mice. Mice were sacrificed 1 week after injection and the nodules formed from HKCs were processed to make frozen blocks with OCT (Fisher Scientific, Hanover Park, IL, USA).

### Chromatin Immunoprecipitation (ChIP)

Human epidermis was separated from the underlying dermis by a brief heat treatment [27]. The finely minced tissue samples were then cross-linked with 1% formaldehyde/PBS at RT for 10 min, followed by addition of 125 mM glycine. After washes in PBS, the tissue pellets were processed for chromatin immunoprecipitation (ChIP) assays as described in [26], using the ChIP assay kit (Millipore) and the rabbit anti-RORα antibody (ab60134, Abcam), in parallel with the affinity-purified non-immune rabbit IgG. The relative amount of precipitated DNA was analyzed by qRT-PCR using primers against the RORE-containing or RORE-negative regions, and calculated after normalization to total input chromatin, according to the formula: % total = 2^ΔCt^×5, where ΔCt = Ct (input) – Ct (immunoprecipitation), Ct, cycle threshold.

### Statistics

All statistical evaluations were carried out using GraphPad Prism 5.0. All analyses are unpaired two-tailed Student’s t-test. Real-time RT-PCR samples were tested in triplicate, and repeated at least three times. After normalization to the housekeeping gene 36β4, combined data was represented as mean-fold over control ± S.E.M. P-values<0.05 were considered significant.

## Supporting Information

Figure S1 **Immunofluorescence analysis of RORα in human skin.** Frozen sections (8 µm) of normal human skin were co-stained with antibodies against RORα (green) and keratin 14 (red). DNA was counterstained with Hoechst (blue). Images are representatives of independent fields from 2 skin samples, derived from different patients, as in Fig. 1D, bar = 50 µm.
(TIF)

Figure S2 **Immunofluorescence analysis of RORα in human skin SCC specimens.** (A–B) Frozen sections (8 µm) of skin SCC samples were stained with the antibody against RORα (green). DNA was counterstained with Hoechst (blue). (A) Top panel: low magnification (10x) of images showing both normal epidermis and SCC lesions from specimen #1. Lower panel: high magnification (20x) of selected areas (a, SCC lesion; b, epidermis) of top panel. (B) High magnification (20x) of RORα staining in epidermis and skin SCC lesion from specimen #2. Bar = 50 µm.
(TIF)

Figure S3 **Silencing of RORα disrupts keratinocyte differentiation** ***in vivo*** **.** Puromycin selected HKCs harboring lentivirus expressing control or RORα shRNAs were injected intradermally into the back skin of NOD/SCID mice as in Fig. 4. Resulting nodules/cysts were collected on day 8 after the injection, and frozen sections were analyzed for expression of K10 (green)/integrin α6 (red) [A], or loricrin (green)/integrin α6 (red) [B]. DNA was counterstained with Hoechst (blue). Shown are the results determined from 4 different mice besides the ones shown in Fig. 4. Upper bar = 100 µm, lower bar = 50 µm.
(TIF)

Figure S4 **RORα does not affect the expression of transcription factors, including p53, c-myc, and NF-κB in HKCs.** HKCs with increased (A) or knocked-down (B) RORα expression were analyzed for expression of individual transcription factors by real time qRT-PCR. Values are presented as mean fold-change over control ± S.E.M, N = 3.
(TIF)

Table S1 **Primer information.**
(PDF)
