# Supplementary figures and images for: The Retinoid-Related Orphan Receptor RORα Promotes Keratinocyte Differentiation via FOXN1
Source: PLoS One. 2013 Jul 29;8(7):e70392. doi: 10.1371/journal.pone.0070392 (PMC3726659; doi:10.1371/journal.pone.0070392)

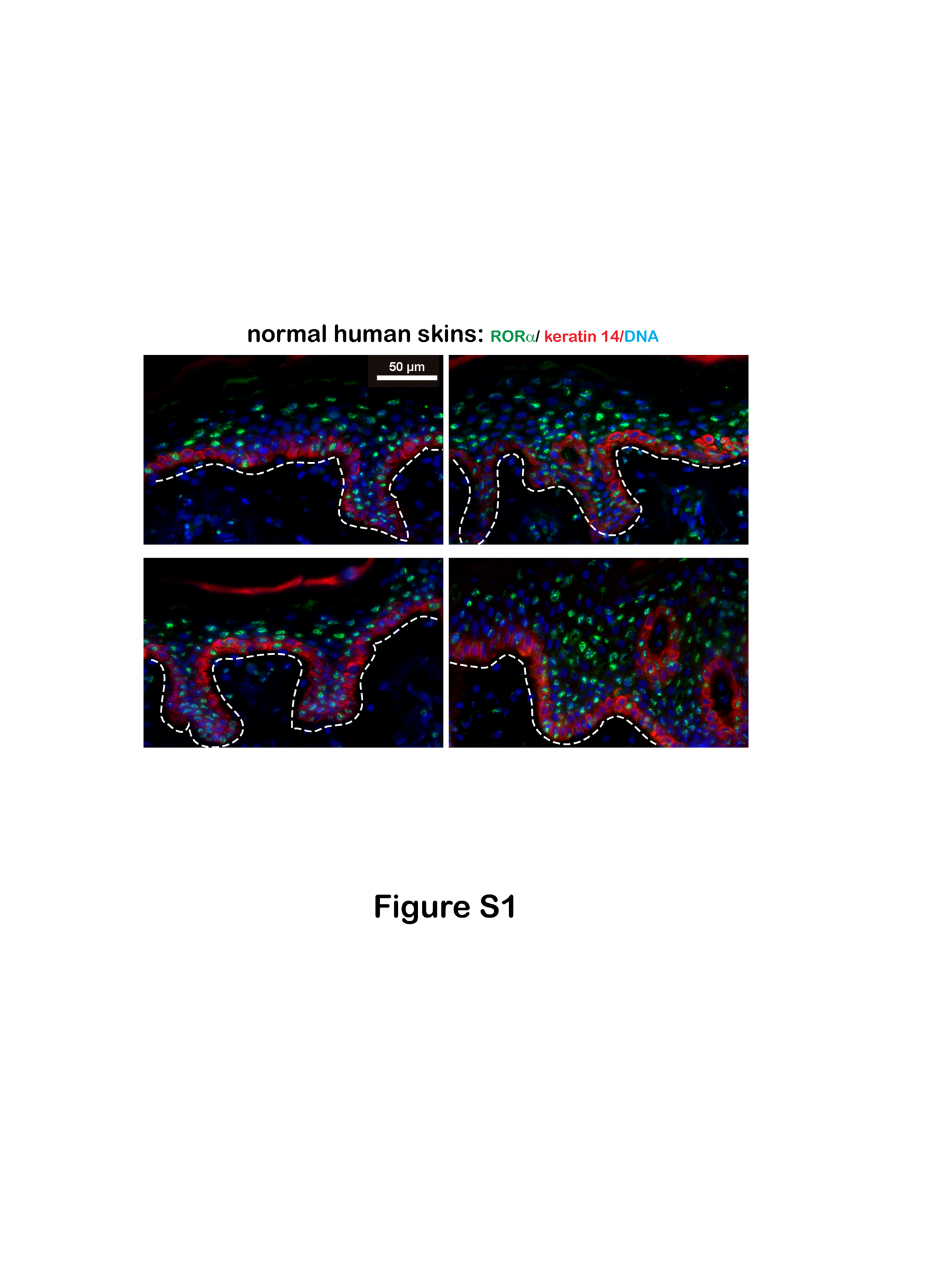

Supplement: Figure S1 — Immunofluorescence analysis of RORα in human skin. Frozen sections (8 µm) of normal human skin were co-stained with antibodies against RORα (green) and keratin 14 (red). DNA was counterstained with Hoechst (blue). Images are representatives of independent fields from 2 skin samples, derived from different patients, as in Fig. 1D, bar = 50 µm. (TIF) [file pone.0070392.s001.tif]

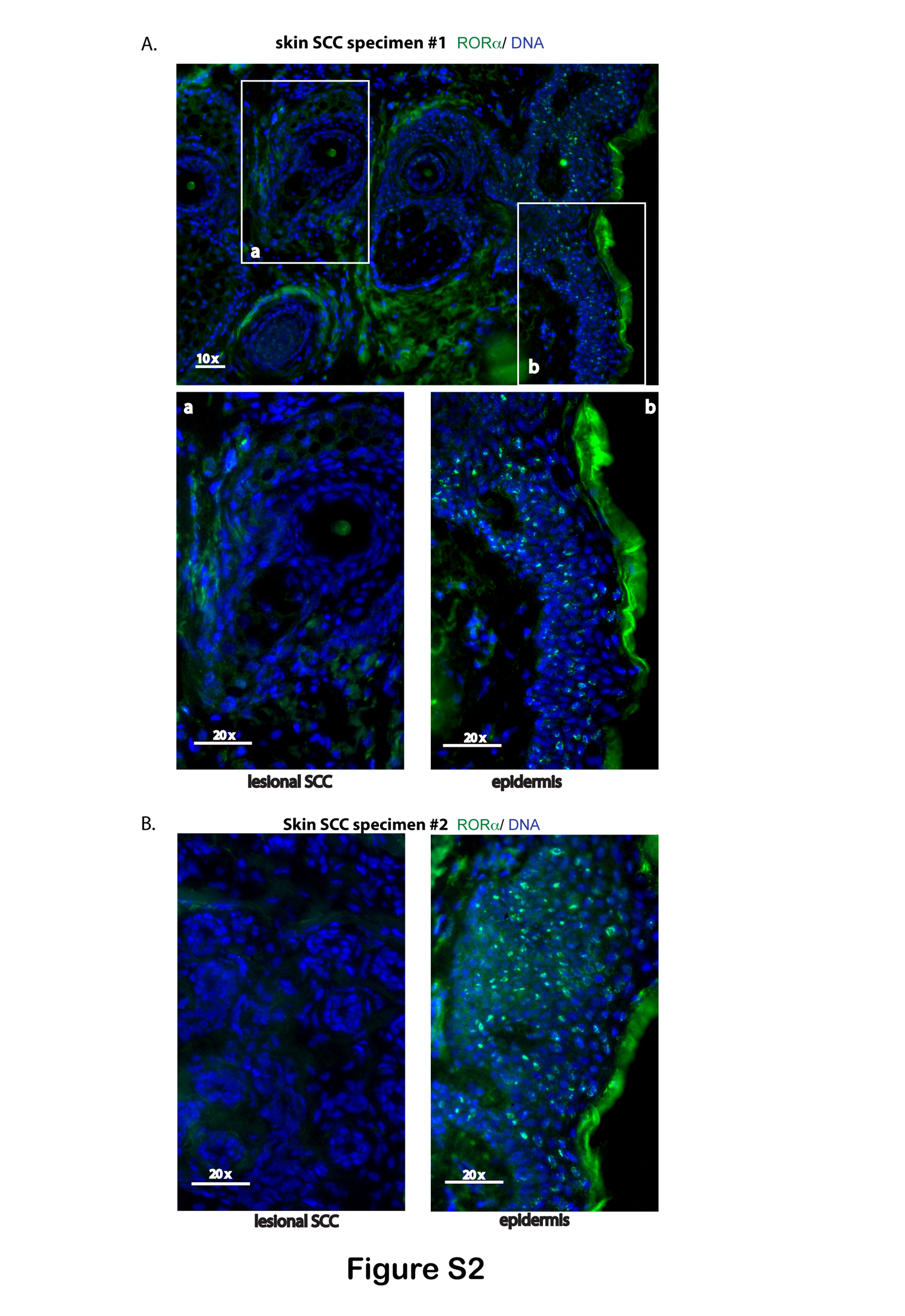

Supplement: Figure S2 — Immunofluorescence analysis of RORα in human skin SCC specimens. (A–B) Frozen sections (8 µm) of skin SCC samples were stained with the antibody against RORα (green). DNA was counterstained with Hoechst (blue). (A) Top panel: low magnification (10x) of images showing both normal epidermis and SCC lesions from specimen #1. Lower panel: high magnification (20x) of selected areas (a, SCC lesion; b, epidermis) of top panel. (B) High magnification (20x) of RORα staining in epidermis and skin SCC lesion from specimen #2. Bar = 50 µm. (TIF) [file pone.0070392.s002.tif]

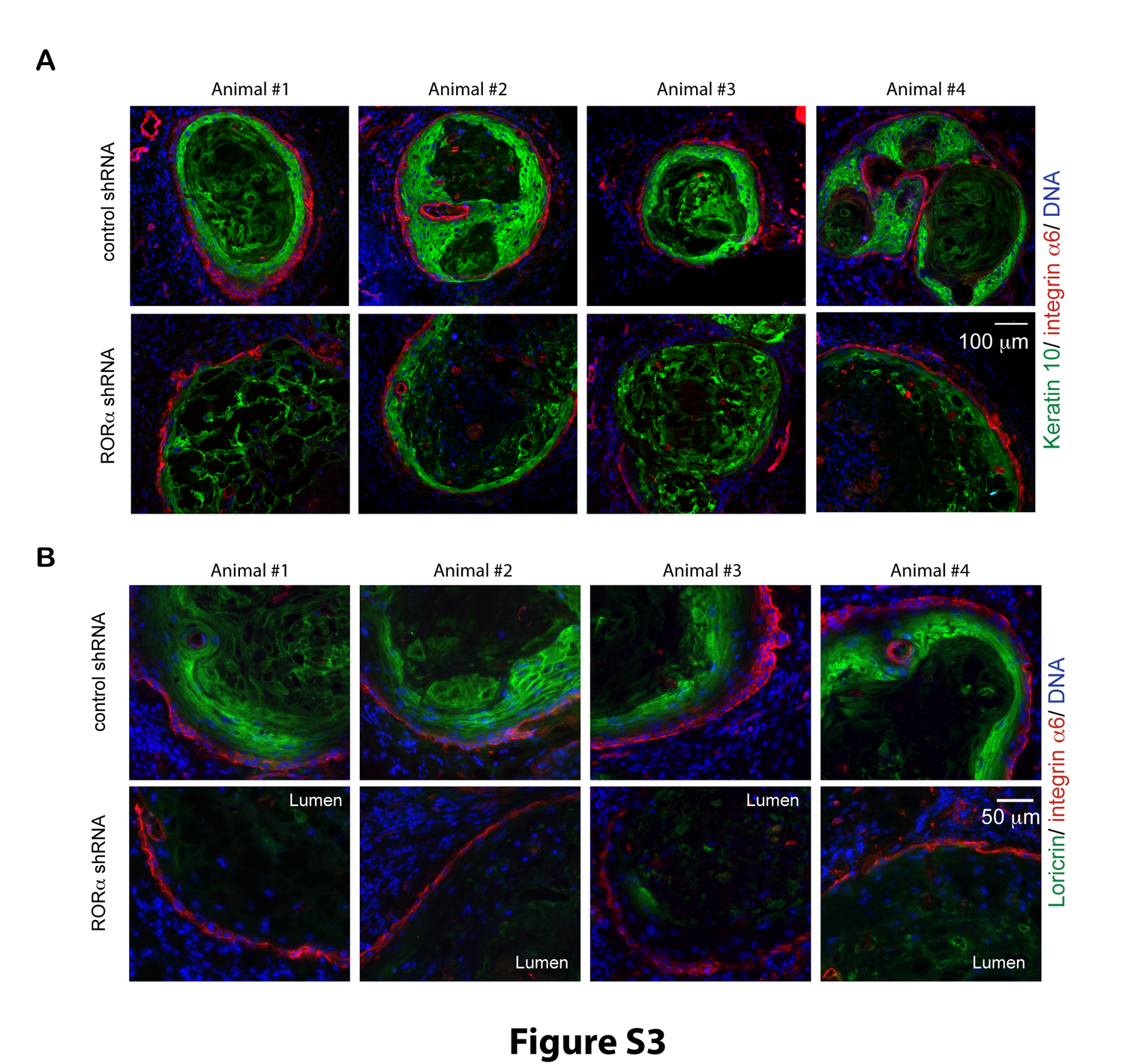

Supplement: Figure S3 — Silencing of RORα disrupts keratinocyte differentiation in vivo . Puromycin selected HKCs harboring lentivirus expressing control or RORα shRNAs were injected intradermally into the back skin of NOD/SCID mice as in Fig. 4. Resulting nodules/cysts were collected on day 8 after the injection, and frozen sections were analyzed for expression of K10 (green)/integrin α6 (red) [A], or loricrin (green)/integrin α6 (red) [B]. DNA was counterstained with Hoechst (blue). Shown are the results determined from 4 different mice besides the ones shown in Fig. 4. Upper bar = 100 µm, lower bar = 50 µm. (TIF) [file pone.0070392.s003.tif]

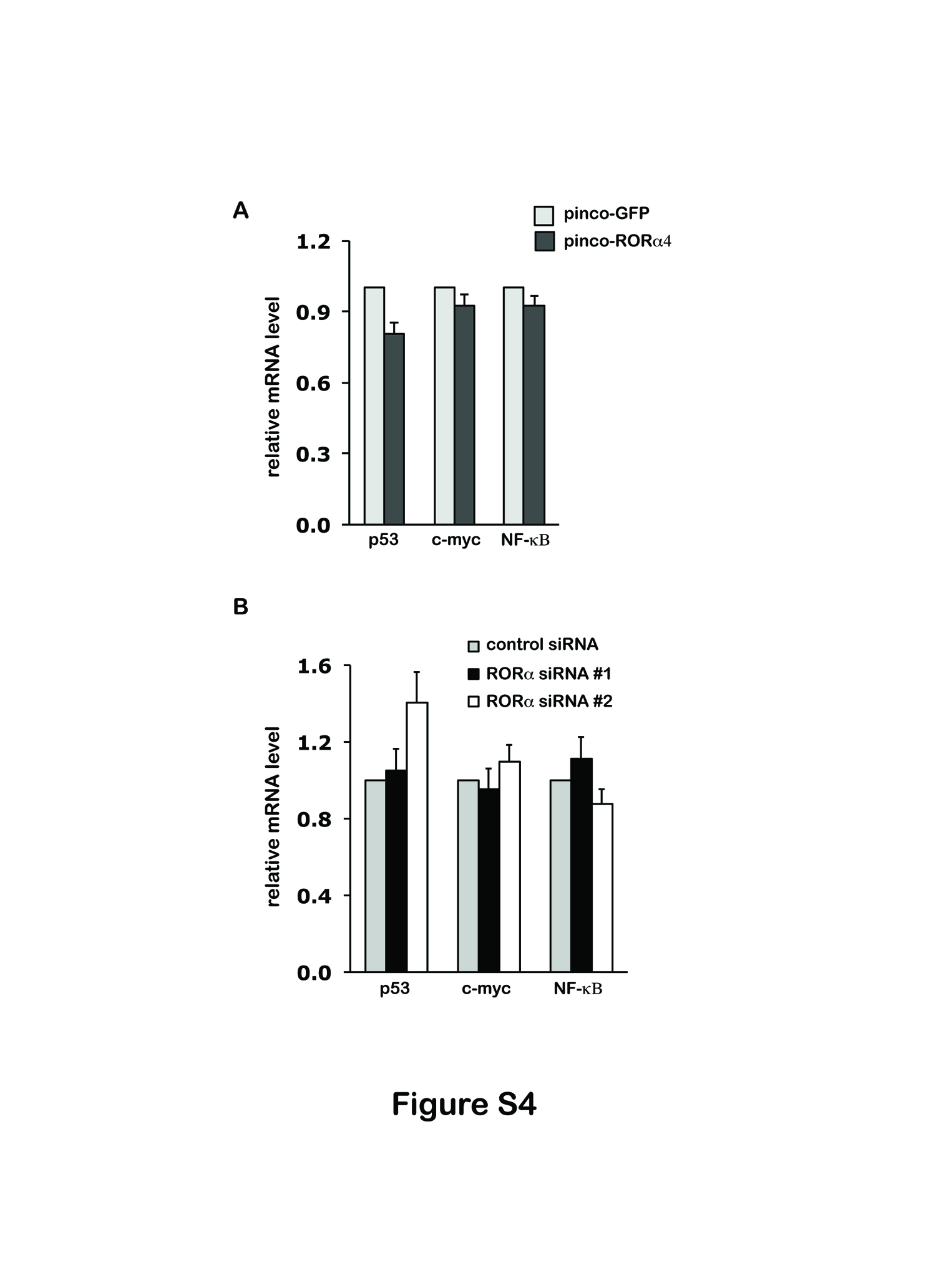

Supplement: Figure S4 — RORα does not affect the expression of transcription factors, including p53, c-myc, and NF-κB in HKCs. HKCs with increased (A) or knocked-down (B) RORα expression were analyzed for expression of individual transcription factors by real time qRT-PCR. Values are presented as mean fold-change over control ± S.E.M, N = 3. (TIF) [file pone.0070392.s004.tif]
