# Supplementary material for: The Retinoid-Related Orphan Receptor RORα Promotes Keratinocyte Differentiation via FOXN1
Source: PLoS One. 2013 Jul 29;8(7):e70392. doi: 10.1371/journal.pone.0070392 (PMC3726659; doi:10.1371/journal.pone.0070392)
Supplement: Table S1 — Primer information. (PDF) [file pone.0070392.s005.pdf]

Table S1

GENE	FORWARD	REVERSE
**Primers for Fig. 1A**		
RORα1	AAACATGGAGTCAGCTCCG	CATACAAGCTGTCTCTCTGC
RORα2	CTCCAAATACTCCATCAGTGTATCC	CATACAAGCTGTCTCTCTGC
RORα3	CAACTTGAGCACATAAACTGG	CATACAAGCTGTCTCTCTGC
RORα4	CGCACCGCGCTTAAATGATG	CATACAAGCTGTCTCTCTGC
**Primers for RT-PCR**		
RORα4	CCGCGGCTTAAATGATGTAT	TTCTCCTGAAAAAGCCCTTG
RORα universal	TGATGTGGCAATTGTGTGC	GCACGGCACATTCTGATAAA
KERATIN 1	GTTCCAGCGTGAGGTTTGTT	TAAGGCTGGGACAAATCGAC
KERATIN 10	GAAAAGCATGGGCAACTCACA	TGTCGATCTGAAGCAGGATG
INVOLUCRIN	GGCCCTCAGATCGTCTCATA	CACCCTCACCCCATTAAAGA
LORICRIN	ATGATGCTACCCAGGTTTG	ACTGGGGTTGGGAGGTAGTT
FILAGGRIN	GGGAAGTTATCTTTTCCTGTC	GATGTGCTAGCCCTGATGTTG
INTEGRIN β4	CCCAACCACTCCTACGTGTT	GGGAGTGCTCAAAGTGAAGG
KERATIN 14	ATATGGTGGTGGCCTTGGTA	GAGGTTCTGCATGGTCACCT
Ki67	CTGCTTGTTTGGAAGGGGTA	AGCCGTACAGGCTCATCAAT
ALOXE3	CCAATCCCCAAACTCCTTCT	CATGTTAGACCCAGGCACAC
ADFP	GAGATGGCAGAGAACGGTGT	AGCCCCTTACAGGCATAGGT
AQP3	AGA CAG CCC CTT CAG GAT TT	CAT ACC TGC TGC CCA TTC TC
ABCA12	TCAATG GCAGTCAAGTGCTC	TCCAAGAGAAGGCATCATCC
36B4	GCAATGTTGCCAGTGTCTGT	GCCTTGACCTTTTCAGCAAG
FOXN1	TTCCCGTCAGCGAGATCTACA	GTTCTCCACCTTCTCGAAGCAC
Notch1	TTGGGAGGAGCAGATTTTTG	CACTGGCATGACACACAACA
primary FOXN1	AGAGTAGGGTCCCAGCCAGT	GTGAGGGTGTCCTCTCTGGA
p53	AGG CCT TGG AAC TCA AGG AT	CTG AGT CAG GCC CTT CTG TC
c-myc	TTCGGGTAGTGGAAAACCAG	GCAGTAGAAATACGGCTGCAC
NF-κB	ACCAGCCTCTGTGTTTGTCC	TGACGTTTCCTCTGCACTTCT
**Primers for ChIP in Fig. 5C**		
FOXN1 promoter -4.8 kb	GCACTGCAGATGTGGAACTGCG	TCACTCTCATGAACTGCCCCTCA
FOXN1 gene + 1.6 kb	TGTCCTTCCATAGCCTGACC	GGGAGCAAATTCCCTTTAGC
FOXN1 RORE negative	CAGGCAGGATAAAGGTTGGA	TCTGGGACTTATGTGGAGAGG
